# [Retracted] Matrine inhibits ovarian cancer cell viability and promotes apoptosis by regulating the ERK/JNK signaling pathway via p38MAPK

**DOI:** 10.3892/or.2024.8758

**Published:** 2024-06-17

**Authors:** Xin Liang, Jianxin Ju

Oncol Rep 45: 82, 2021; DOI: 10.3892/or.2021.8033

Following the publication of the above article, a concerned reader drew to the Editor's attention that certain of the Transwell cell invasion assay data featured in Fig. 1B and C, the immunofluorescence assay data in Fig. 2E and F, the TUNEL assay data in Fig. 4C and the immunohistochemical data in Fig. 4B and E were strikingly similar to data appearing in different form in other articles written by different authors at different research institutes that had either already been published elsewhere prior to the submission of this paper to *Oncology Reports*, or which under consideration for publication at around the same time.

In view of the fact that certain of these data had already apparently been published prior to the submission of this article for publication, the Editor of *Oncology Reports* has decided that this paper should be retracted from the Journal. The authors were asked for an explanation to account for these concerns, but the Editorial Office did not receive a reply. The Editor apologizes to the readership for any inconvenience caused.

